# Enhanced Macular Telangiectasia Type 2 Detection: Leveraging Self-Supervised Learning and Ensemble Models

**DOI:** 10.1016/j.xops.2025.100710

**Published:** 2025-01-13

**Authors:** Shahrzad Gholami, Lea Scheppke, Meghana Kshirsagar, Yue Wu, Rahul Dodhia, Roberto Bonelli, Irene Leung, Ferenc B. Sallo, Alyson Muldrew, Catherine Jamison, Tunde Peto, Juan Lavista Ferres, William B. Weeks, Martin Friedlander, Aaron Y. Lee, Lowy Medical Research Institute

**Affiliations:** 1 AI for Good Research Lab, Microsoft, Redmond, Washington; 2 The Lowy Medical Research Institute, La Jolla, California; 3 Department of Ophthalmology, University of Washington, Seattle, Washington; 4 Roger and Angie Karalis Johnson Retina Center, Seattle, Washington; 5 Moorfields Eye Hosptial NHS Foundation Trust, London, United Kingdom; 6 Department of Ophthalmology, Jules Gonin Eye Hospital, University of Lausanna, Lausanna, Switzerland; 7 Centre for Public Health, Queen's University Belfast, Belfast, Northern Ireland, United Kingdom; 8 Department of Cellular and Molecular Biology, the Scripps Research Institute, La Jolla, California

**Keywords:** Macular telangiectasia type 2, Deep learning, Ensemble models, Self-supervised learning, OCT imaging

## Abstract

**Objective:**

To investigate an ensemble-based approach utilizing deep learning models for accurate and interpretable detection of macular telangiectasia (MacTel) type 2 on OCT imaging.

**Design:**

Retrospective analysis of OCT scans, model development, and assessment.

**Participants:**

A total of 5200 OCT images from participants in the MacTel Registry conducted by the Lowy Medical Research Institute and from the University of Washington (780 MacTel patients and 1900 non-MacTel patients).

**Methods, Intervention, or Testing:**

We trained multiple individual MacTel vs. non-MacTel classification models using traditional supervised learning and self-supervised learning (SSL) and ensembled them using average weighting methods. We investigated diverse methodologies for constructing the ensemble, including varied architectural configurations and learning paradigms of individual models, and manipulating the amount of labeled data accessible for training. Model performance was compared against human expert graders on held-out test set data. Model interpretability was investigated using gradient-weighted class activation maps (Grad-CAM) visualization and by evaluating interrater agreement.

**Main Outcome Measures:**

For model performance, area under the receiver operating characteristic curve (AUROC), area under the precision–recall curve (AUPRC), accuracy, sensitivity, and specificity were reported. For interpretability, interrater agreements and Grad-CAM visualization results were evaluated.

**Results:**

Despite access to only 419 OCT volumes, including 185 MacTel patients within the 10% labeled training dataset, the ensemble model demonstrated a performance level (AUROC 0.972 [95% confidence interval (CI), 0.971–0.973], AUPRC 0.967 [95% CI, 0.965–0.969], accuracy 91.7%, sensitivity 0.905, and specificity 0.925) comparable to the human experts ensemble (AUROC 0.977 [95% CI, 0.975–0.978], AUPRC 0.987 [95% CI, 0.986–0.987], accuracy 96.8%, sensitivity 0.929, and specificity 1) on a test set of 500 patients. The individual models did not achieve the same performance levels when evaluated separately.

**Conclusions:**

Even with limited data, combining SSL with ensemble approaches improved MacTel classification accuracy and interpretation compared to the individual models. Self-supervised learning captures meaningful representations from unlabeled data, a key benefit in the setting of limited data such as with rare diseases.

**Financial Disclosure(s):**

Proprietary or commercial disclosure may be found in the Footnotes and Disclosures at the end of this article.

Macular telangiectasia (MacTel) is a retinal disease historically challenging to diagnose and often subject to misidentification. However, increased awareness has led to improved diagnostic outcomes.^1^ MacTel diagnosis relies upon a multimodal image set and the expertise of clinicians familiar with the disease. OCT imaging has emerged as a valuable tool for the diagnosis and monitoring of various retinal diseases.^2^^,^^3^ With the increasing integration of OCT into clinical practice,^4^ deep learning models may be able to achieve accurate MacTel prediction comparable to that of retinal specialists, even when working with limited data. Moreover, the insights garnered from these models hold the potential to significantly advance research in this domain, as the utilization of deep learning for MacTel classification has hitherto been underexplored due to data constraints, mainly because of the disease's rarity.^5^ In this study, we focus on the accurate classification of MacTel type 2 using OCT images, with the overarching goal of facilitating early and precise detection of this neurodegenerative disease.^6^

Previous studies have successfully employed ensemble techniques for various medical imaging tasks, such as diabetic retinopathy diagnosis from fundus images^7^ and breast cancer detection.^8^ Ensembling is designed to mitigate the bias of each strong classifier and leverage the diversity of individual machine learning models within the ensembles.^9^^,^^10^ Deep learning ensemble models have even outperformed human expert ensembles in other domains.^11^ To address the problem of limited data, and building upon these insights in the context of medical imaging, we aimed to investigate an ensemble approach for enhancing the accuracy and interpretability of MacTel classification models when dealing with limited labeled data for training. To accomplish this goal, we leveraged the ResNet18 and ResNet50 neural network architectures, encompassing both traditional supervised and self-supervised learning (SSL) paradigms as introduced in prior work.^12^ Our approach involves the training of multiple individual models and their assembly into ensembles utilizing average weighting concept. Our investigative framework explores various methodologies for constructing the ensemble, including variations in architectural configurations, learning paradigms, and adjustments to the volume of labeled data accessible for neural network training. We aimed to compare model performance to human expert graders, as well as to explore visualization techniques to identify the regions within the OCT images that contribute to the models' predictions.

We investigated this novel ensemble approach for automated MacTel classification on OCT imaging, employing both traditional supervised and SSL, with the goal of better addressing the limited data challenges associated with rare diseases like MacTel.

## Methods

### OCT Image Dataset

We use datasets obtained using a Spectralis OCT device, collected through the MacTel Project Natural History and Observation Registry Study,^13^ which includes 2636 OCT scans from 780 MacTel patients and 131 non-MacTel patients. The dataset was augmented with an additional 2564 scans of 1769 non-MacTel patients, which were continuously collected (all patients with a routinely collected macular OCT were included) between 2006 and 2016 at the University of Washington (UW). This study was approved by central or local institutional review boards associated with the MacTel Registry study and by the UW Institutional Review Board and is in adherence with the tenets of the Declaration of Helsinki. All participants provided written informed consent to participate. Table 1, Table 2 present the racial distributions for the UW and MacTel datasets. The UW dataset includes a mean age of 61.24 years (standard deviation = 18.09), with a sex distribution of 53% female and 47% male. The MacTel dataset includes a mean age of 60.76 years (standard deviation = 11.66) and a sex distribution of 64% female and 36% male.

**Table 1:** Race Distribution for UW Dataset

Race	Percentage
White	63.3%
Asian	13.7%
Black or African American	11.1%
Unavailable or unknown	5.4%
American Indian or Alaska Native	1.7%
Native Hawaiian or other Pacific Islander	1.2%
Declined to answer	3.6%
Mexican, Mexican-American, or Chicano	0.0%
Laotian	0.0%
Patient not present	0.0%
NULL	0.0%
Unknown	0.0%

UW = University of Washington.

**Table 2:** Race Distribution for MacTel Dataset

Race	Percentage
White	91%
American Indian or Alaskan Native	1%
Asian	3%
Black/African	1%
Native Hawaiian or Pacific Islander	0%
No response/no data	0%
Other	3%

MacTel = macular telangiectasia.

**Figure 1:**
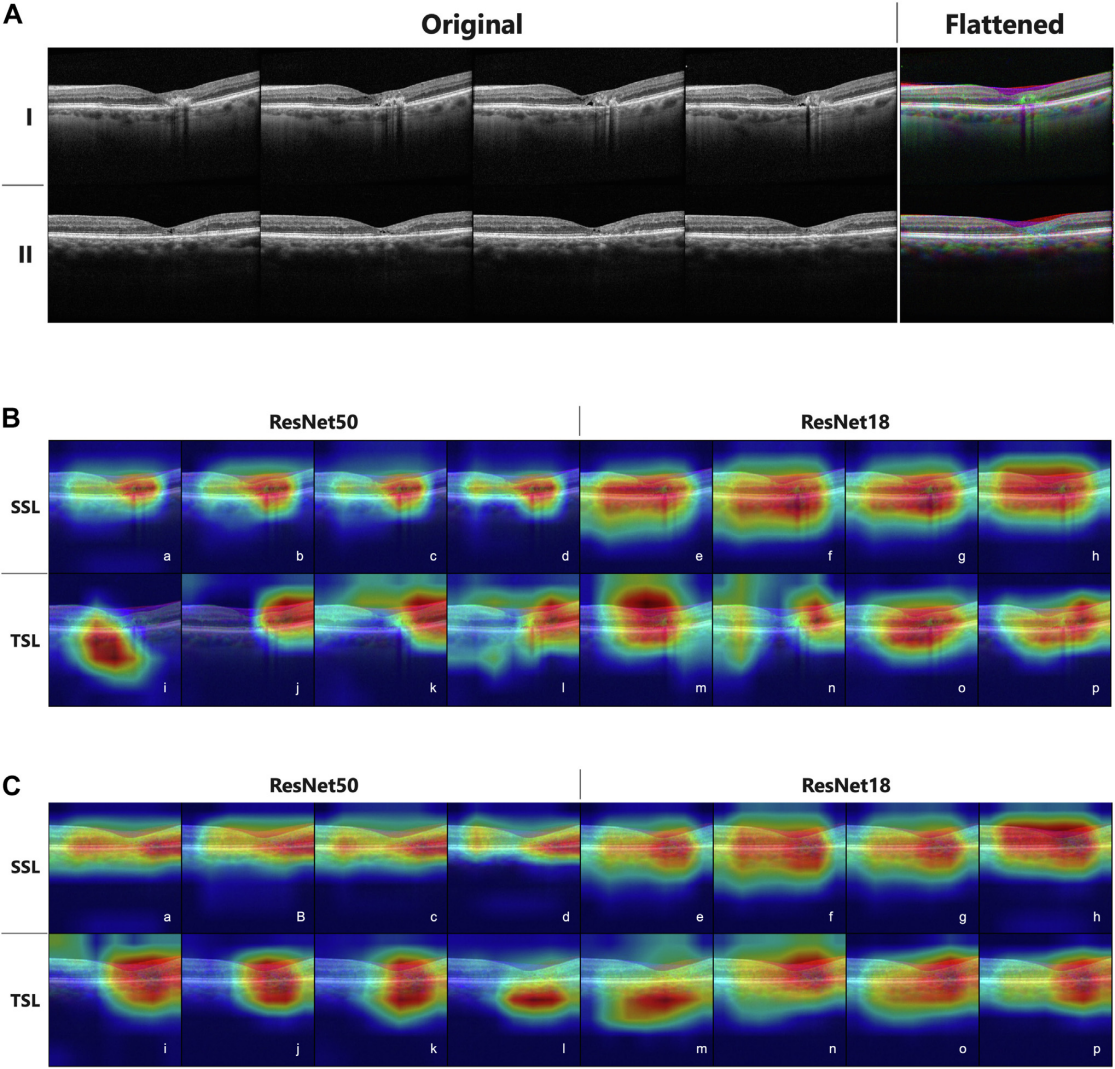
OCT images (**A**) and the corresponding Grad-CAM results for individual models for 2 patients with MacTel (**B,C**); in Grad-CAM results, a–d show results for individual models learned based on various amounts of labeled data from 10% to 100% for ResNet50 using the self-supervised learning (SSL) approach, e–h show results for ResNet18 using the SSL approach, i–l shows results for ResNet50 using the traditional supervised learning (TSL) approach, and m–p show results for ResNet18 using the TSL approach. Grad-CAM = gradient-weighted class activation maps; MacTel = macular telangiectasia.

The dimensions of the OCT volumes varied in terms of width and height. To ensure uniformity in sample size, all volumes were resampled to a fixed dimension of 496 × 768 × 196 B-scans using linear interpolation. To streamline computations and concentrate on disease-relevant areas, we selected the central third of B-scans from each volume and resampled them into 3 B-scans. These 3 B-scans were then combined to form a red, green, and blue image with 3 channels, where each channel represented a single B-scan. This flattening approach enabled us to leverage contextual information from neighboring B-scans in 2-dimensional neural network architectures. Figure 1A illustrates several OCT B-scans along with flattened versions for a MacTel patient in the first row and a non-MacTel patient in the second row, focusing on the region around the fovea. The resulting dataset consisted of 5200 volumes. To enhance the model's robustness, we applied data augmentation techniques such as random horizontal flips and center crops. We randomly divided the dataset into training, validation, and test sets, with an 80:10:10 ratio. The training set consists of 2348 positive and 1852 negative samples. The validation set consists of 262 positive and 238 negative samples, and the test set consists of 225 positive and 275 negative samples. The training and validation sets were used for model training and hyperparameter tuning, while the test set was reserved for the final model performance evaluation. We do not have access to any additional grading information showing the stage of MacTel; thus, our study focuses on MacTel vs. non-MacTel classification of the disease.

### Deep Learning Models Training

We trained ResNet18 and ResNet50 architectures using established traditional supervised learning (TSL) and SSL approaches.^12^ These models were implemented in PyTorch and optimized using stochastic gradient descent with a learning rate of 0.001 and a batch size of 32. We initialized the models' weights with pretrained weights from ImageNet, a vast dataset of natural images. Subsequently, we fine-tuned these weights on our OCT dataset for 100 epochs, employing early stopping based on the binary cross-entropy loss observed on the validation set.

We trained the individual models on different percentages of labeled data based on the TSL or SSL approaches as described previously in prior work.^12^

### Ensembling Approach

To enhance the performance of our deep learning models, we explored the application of ensemble methods on OCT images. We conducted experiments using uniform averaging of individual models' predicted probability outputs as well as the AdaBoost algorithm to combine the results of selected individual deep learning models (DL-Adaboost)^14^^,^^15^ (see Supplemental Appendix, available at www.ophthalmologyscience.org). Unlike uniform averaging of models, where the ensemble is formed based on averaging the predictions, DL-AdaBoost iteratively adjusts the weights assigned to each model based on their performance, aiming to minimize the weighted error.^11^^,^^16^ We applied both ensemble methods on the model's predicted probabilities and observed no significant difference in accuracy metrics between DL-Adaboost and uniform averaging. We present uniform averaging results in the Results section, with DL-Adaboost results detailed in the Supplemental Appendix. Prior work also found that uniform averaging tends to perform comparably to more complex ensemble strategies in many applications, supporting it as a reliable default.^17^ However, it is worth noting that ensemble averaging can be ineffective when models are highly complementary, as conflicting predictions may cancel each other out, reducing accuracy.^18^ Additionally, if models produce nearly identical outputs, averaging offers minimal improvement, as it reflects the same biases and lacks additional information.^17^

We constructed 17 different ensemble models using various combinations of the above individual models to assess the impact of each model training configuration. The first set of ensembles was formed based on individual models trained via the TSL approach. They are named RESNET50-ENSM(TSL), RESNET18-ENSM(TSL), and RESNET(50&18)-ENSM(TSL) based on the architecture(s) and methods used for model training. For example, in RESNET50-ENSM(TSL), only individual models, which are trained based on the ResNet50 architecture, utilize the uniform averaging approach. RESNET(50&18)-ENSM(TSL) incorporates a combination of ResNet18 and ResNet50 architecture-based models. Note that in naming the models, we used RESNET(50&18) to indicate the case where individual models based on both architectures are used in the ensemble. The second set of ensembles is similar to the previous set, except that we used individual models based on the SSL approach. The third set of ensembles is formed using models trained based on SSL and TSL approaches. In the fourth set of ensembles, we fix the percentage of labeled training data used to train individual models based on different architectures and learning paradigms. This case is specifically important in the context of rare diseases when limited data are available for model training. For the final set of models, we split the training data into 4 nonoverlapping subsets and independently trained models using either SSL or TSL for a single architecture choice. We then created ensembles from these models; "4X" indicates that each ensemble consists of 4 independently trained models, each trained on 25% of the training data.

Weights are assigned uniformly to each individual model within the ensemble for all scenarios.

### Gradient-Weighted Class Activation Map

We utilized gradient-weighted class activation maps (Grad-CAM) inferred from the global pooling layer of the deep learning models, which provide a visual representation, resembling a thermogram, highlighting suspected pathology within the image.^19^ The global pooling layer aggregates feature maps from the entire spatial extent of the preceding convolutional layers. We also combined Grad-CAMs of the individual models based on the uniformly assigned weights to capture the collective contributions of the individual models in the ensemble.

### Outcome Measures

To gauge the performance of our trained models, we computed several metrics, including accuracy, sensitivity, specificity, area under the receiver operating characteristic curve (AUROC), and area under the precision–recall curve (AUPRC), on the reserved test set.

We compared the performance of our models with that of 4 individual human graders and to an ensemble created from their assessments, as detailed in prior work.^12^ We compared the performance of our models to that of 4 expert graders. Graders 1 and 2 had extensive MacTel grading experience (14–17 years), whereas Graders 3 and 4 were less experienced MacTel graders (<2 years). The human expert ensemble employed a voting mechanism among the 4 graders; a positive diagnosis was recorded when at least 2 graders reported a positive assessment. We employed Cohen kappa score to assess the alignment among the individual and ensemble human graders and among our individual and ensemble neural network models.

## Results

### Individual Models

The test performance of individual models, ResNet50 and ResNet18, trained on different percentages of labeled data using the TSL or SSL approaches, is shown in Table 3. These individual models are trained to be potentially used as individual contributors to the ensemble models. Both architectures exhibit satisfactory performance in MacTel classification. However, as the amount of labeled data increases, the performance of ResNet18 models consistently improves in comparison to ResNet50. This trend may be attributed to the higher susceptibility of ResNet50, which has approximately 23 million parameters, to overfitting, unlike ResNet18. Also, as shown in prior work,^12^ pretraining based on SSL improved the model performance.

**Table 3:** Test Set Performance of Individual Models Trained on Various Amounts of Labeled Data for ResNet50 and ResNet18 and Trained Based on Either Traditional Supervised Learning (TSL) or Self-Supervised Learning (SSL) Approach

TAG	Architecture	% of Labels	AUROC (CI 95%)	AUPRC (CI 95%)	Accuracy	Sensitivity	Specificity
A	RESNET50 (SSL)	10%	0.951 (0.949–0.953)	0.942 (0.939–0.944)	0.845	0.949	0.761
B		25%	0.968 (0.967–0.970)	0.967 (0.965–0.969)	0.892	0.951	0.845
C		50%	0.967 (0.965–0.968)	0.961 (0.960–0.963)	0.893	0.952	0.846
D		100%	0.971 (0.969–0.972)	0.972 (0.971–0.974)	0.926	0.899	0.948
E	RESNET18 (SSL)	10%	0.964 (0.963–0.966)	0.958 (0.957–0.960)	0.899	0.882	0.914
F		25%	0.975 (0.974–0.976)	0.969 (0.967–0.971)	0.91	0.945	0.881
G		50%	**0.976 (0.974–0.977)**	**0.971 (0.969–0.973)**	0.908	0.959	0.866
H		100%	0.971 (0.969–0.972)	0.965 (0.963–0.966)	0.895	0.948	0.853
I	RESNET50 (TSL)	10%	0.947 (0.945–0.949)	0.939 (0.936–0.941)	0.884	0.844	0.917
J		25%	0.958 (0.956–0.959)	0.953 (0.951–0.955)	0.896	0.864	0.923
K		50%	0.936 (0.934–0.938)	0.933 (0.931–0.935)	0.869	0.892	0.85
L		100%	0.947 (0.945–0.949)	0.935 (0.932–0.938)	0.898	0.899	0.896
M	RESNET18 (TSL)	10%	0.853 (0.850–0.856)	0.839 (0.834–0.843)	0.762	0.839	0.699
N		25%	0.942 (0.940–0.944)	0.923 (0.919–0.926)	0.859	0.763	0.937
O		50%	0.948 (0.946–0.950)	0.946 (0.944–0.948)	0.867	0.93	0.815
P		100%	0.965 (0.963–0.966)	0.963 (0.962–0.965)	0.89	0.876	0.902
G1	Grader 1	-	-	-	0.95	0.902	0.989
G2	Grader 2	-	-	-	0.95	0.893	0.996
G3	Grader 3	-	-	-	0.91	0.8	1
G4	Grader 4	-	-	-	0.88	0.747	0.989

AUPRC = area under the precision-recall curve; AUROC = area under receiver operating characteristic; CI = confidence interval.

Bold text represents the highest achieved AUPRC and AUROC.

### Ensemble Models

Table 4 displays the performance results of the ensemble models on the test set. The ensemble method effectively enhances the overall model performance across the majority of evaluated metrics when comparing results shown in Table 3, Table 4, highlighting the advantages of incorporating multiple individual models within an ensemble framework. Additionally, ensembles formed based on SSL (models D-F) perform better than the ones formed based on TSL (models A-C), based on AUROC, AUPRC, and sensitivity. Furthermore, forming ensemble models using individual models trained based on both TSL and SSL training approaches (models G-I) outperforms a single training approach (models A-F). In all cases, using 2 architectures improves the results. Models J-M show that using 2 architectures and 2 training approaches resulted in robust performance even with a smaller amount of labeled data available.

**Table 4:** Test Set Performance of Ensembles Based on Uniform Averaging; the First Part of the Models' Names Indicates the Architectures Used in Each Ensemble

Tag	Ensemble Model	AUROC (CI 95%)	AUPRC (CI 95%)	Accuracy	Sensitivity	Specificity
A	RESNET50-ENSM(TSL)	0.966 (0.965–0.968)	0.965 (0.964–0.967)	0.912	0.896	0.925
B	RESNET18-ENSM(TSL)	0.968 (0.966–0.970)	0.960 (0.958–0.963)	0.917	0.904	0.927
C	RESNET(50&18)-ENSM(TSL)	0.977 (0.975–0.978)	0.974 (0.972–0.975)	0.929	0.911	0.944
D	RESNET50-ENSM(SSL)	0.970 (0.969–0.972)	0.969 (0.967–0.970)	0.903	0.942	0.872
E	RESNET18-ENSM(SSL)	0.975 (0.974–0.976)	0.970 (0.968–0.972)	0.91	0.945	0.881
F	RESNET(50&18)-ENSM(SSL)	0.978 (0.977–0.979)	0.976 (0.975–0.977)	0.919	0.956	0.888
G	RESNET50-ENSM(SSL-TSL)	0.978 (0.977–0.979)	0.978 (0.977–0.979)	0.939	0.947	0.932
H	RESNET18-ENSM(SSL-TSL)	0.977 (0.976–0.979)	0.974 (0.972–0.975)	0.93	0.94	0.922
I	RESNET(50&18)-ENSM(SSL-TSL)	0.982 (0.981–0.983)	0.982 (0.981–0.983)	0.938	0.956	0.923
J	RESNET(50&18)-ENSM(SSL-TSL)-100%	0.980 (0.979–0.981)	0.980 (0.979–0.981)	0.931	0.938	0.925
K	RESNET(50&18)-ENSM(SSL-TSL)-50%	0.977 (0.976–0.978)	0.975 (0.974–0.977)	0.922	0.962	0.889
L	RESNET(50&18)-ENSM(SSL-TSL)-25%	0.980 (0.979–0.981)	0.977 (0.976–0.979)	0.932	0.944	0.923
M	RESNET(50&18)-ENSM(SSL-TSL)-10%	0.972 (0.971–0.973)	0.967 (0.965–0.969)	0.917	0.905	0.925
N	Human Expert Ensemble	0.977 (0.975–0.978)	0.987 (0.986–0.987)	0.968	0.929	1

AUPRC = area under the precision-recall curve; AUROC = area under receiver operating characteristic; CI = confidence interval; SSL = self-supervised learning; TSL = traditional supervised learning.

(50&18) shows that both architectures are used in the ensemble. Training approaches used for individual models are shown in parentheses. The % number in the models' names shows the particular percentage of labeled data used in the training of individual models.

The ensemble model M that was trained with 10% labeled data demonstrates a level of performance (AUROC 0.972 [95% confidence interval (CI), 0.971–0.973], AUPRC 0.967 [95% CI, 0.965–0.969], accuracy 0.917, sensitivity 0.905, and specificity 0.925) comparable to that achieved by human experts ensemble (AUROC 0.977 [95% CI, 0.975–0.978], AUPRC 0.987 [95% CI, 0.986–0.987], accuracy 0.968, sensitivity 0.929, and specificity 1). In contrast, the individual models a, e, i, and m trained on similar amounts of data did not show similar performances. Notably, the human expert ensemble used as a reference in this study included graders 1 and 2, who were retina specialists with extensive MacTel grading experience (14–17 years), whereas graders 3 and 4 each had less than 2 years of experience.

The ensemble model J that was trained with 100% labeled data showed better performance (AUROC 0.980 [95% CI, 0.979–0.981], AUPRC 0.980 [95% CI, 0.979–0.981], accuracy 0.931, sensitivity 0.938, and specificity 0.925) than the human expert ensemble.

For individual models trained with the full dataset (100%) as presented in Table 3, we observe greater variability in performance across metrics. In contrast, the ensemble model L trained on only 25% of the data in Table 4 shows improved performance considering the range of individual models' performance, trained on 100% of the data, considering all metrics. In particular, we observe AUROC of 0.980 for the ensemble L (compared to range 0.947–0.971 for individual models), AUPRC of 0.977 (compared to range 0.935–0.972), accuracy of 0.932 (compared to range 0.89–0.926), sensitivity of 0.944 (compared to range 0.876–0.948), and specificity of 0.923 (compared to range 0.853–0.948). These findings indicate that, despite access to only one-quarter of the dataset, the ensemble model consistently outperforms the worst-case performance of individual models and, in several instances, even exceeds the best-case performance of those models trained on 100% of the data across all metrics.

Table 5 presents results using only 25% of the data, where the training data were randomly divided into 4 nonoverlapping subsets to construct ensembles based on a single learning paradigm and a single architecture. As also demonstrated in a prior study,^12^ SSL with ResNet18 shows more consistent performance improvements, likely due to the model's size being more compatible with the dataset volume. Our experiments further demonstrate that SSL outperforms TSL for ResNet18 across most metrics, as observed by comparing models Q and R. While ensemble models similar to models O, P, Q, and R enhance performance on certain metrics, broader and more consistent improvement across all metrics is achieved by incorporating additional individual models in the ensemble, as reflected in model L.

**Table 5:** Test Set Performance of Ensembles Based on Uniform Averaging; the First Part of the Models' Names Indicates the Architectures Used in Each Ensemble

Tag	Ensemble Model	AUROC (CI 95%)	AUPRC (CI 95%)	Accuracy	Sensitivity	Specificity
B	RESNET50 (SSL)-25%	0.968 (0.967–0.970)	0.967 (0.965–0.969)	0.892	0.951	0.845
P	RESNET50-4X (SSL)-25%	0.966 (0.965–0.968)	0.964 (0.962–0.966)	0.904	0.916	0.895
I	RESNET50 (TSL)-25%	0.958 (0.956–0.959)	0.953 (0.951–0.955)	0.896	0.864	0.923
R	RESNET50-4X (TSL)-25%	0.971 (0.970–0.973)	0.969 (0.969–0.971)	0.931	0.95	0.915
F	RESNET18 (SSL)-25%	0.975 (0.974–0.976)	0.969 (0.967–0.971)	0.91	0.945	0.881
Q	RESNET18-4X (SSL)-25%	0.979 (0.978–0.980)	0.977 (0.976–0.978)	0.922	0.96	0.891
N	RESNET18 (TSL)-25%	0.942 (0.940–0.944)	0.923 (0.919–0.926)	0.859	0.763	0.937
S	RESNET18-4X (TSL)-25%	0.972 (0.971–0.974)	0.968 (0.966–0.969)	0.921	0.912	0.928
**L**	**RESNET (50&18)-ENSM (SSL-TSL)-25%**	**0.980 (0.979–0.981)**	**0.977 (0.976–0.979)**	**0.932**	**0.944**	**0.923**

AUROC = area under receiver operating characteristic; AUPRC = area under the precision-recall curve; CI = confidence interval; SSL = self-supervised learning; TSL = traditional supervised learning.

Training approaches used for individual models are shown in parentheses. The % number in the models' names shows the particular percentage of labeled data used in the training of individual models. 4X means 4 independently trained models based on 25% of training data are used in ensembles. Bold text represents the highest achieved AUROC and AUPRC.

### Comparison with Human Graders

**Figure 2:**
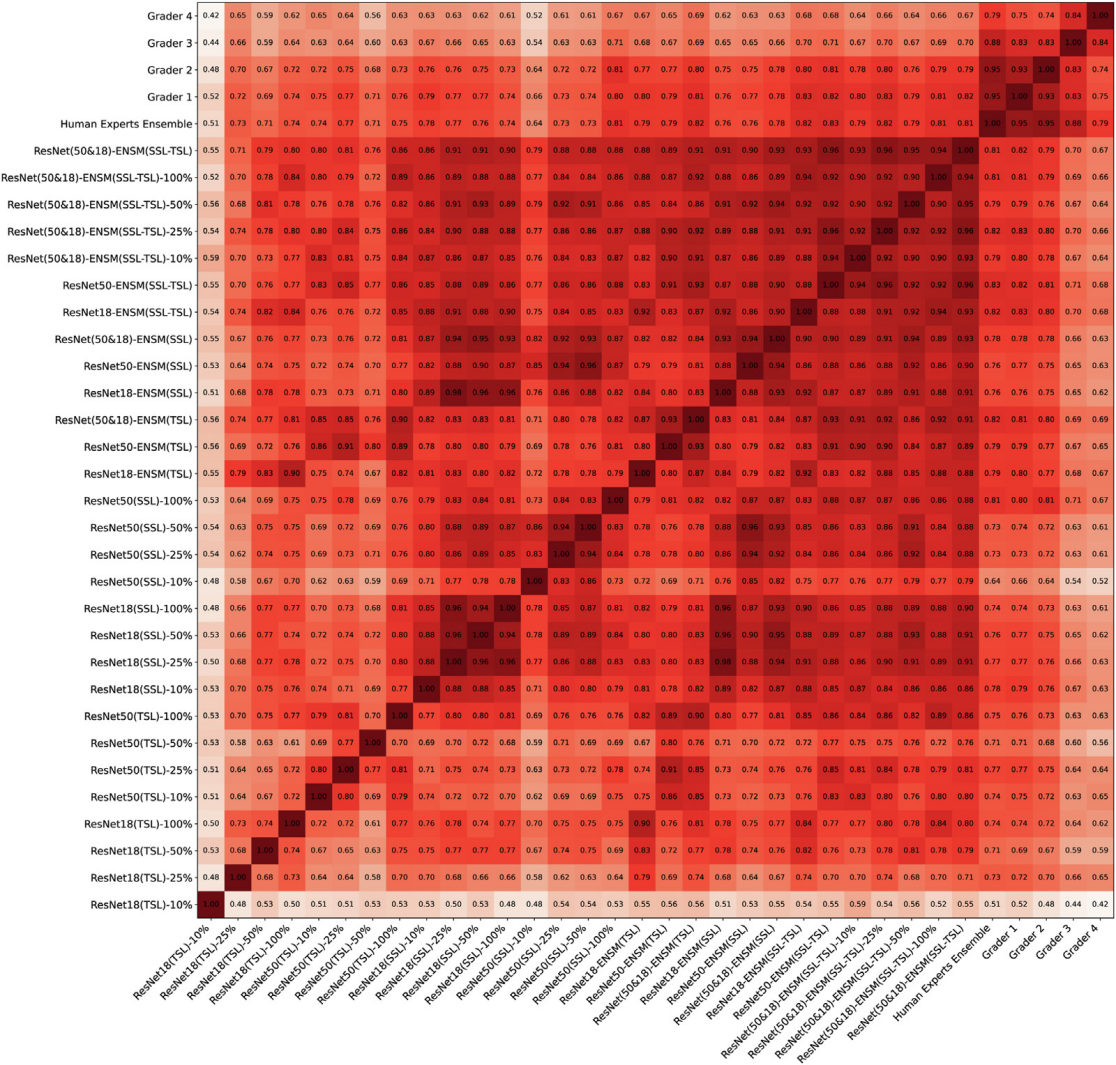
Cohen Kappa Matrix reflects on the interrater agreements based on uniform averaging. We compare agreement between deep learning individual and ensemble models as well as graders. Graders 3 and 4 have less agreement with other graders and deep learning models due to less experience with grading OCT images for MacTel. Models trained based on self-supervised learning show better alignment with the most expert graders, i.e., graders 1 and 2. Ensemble models show better agreement to human expert graders than individual deep learning models. MacTel = macular telangiectasia.

The Cohen kappa matrix, depicted in Figure 2, provides insights into interrater agreements. Notably, graders 3 and 4 exhibited lower levels of agreement with other graders and deep learning models, likely due to their relatively less experience in grading OCT images for MacTel compared with graders 1 and 2. Interestingly, models trained using SSL displayed stronger alignment with relatively more experienced graders, graders 1 and 2. Furthermore, ensemble models demonstrated superior agreement with human expert graders when compared with individual deep learning models.

### Explainability

**Figure 3:**
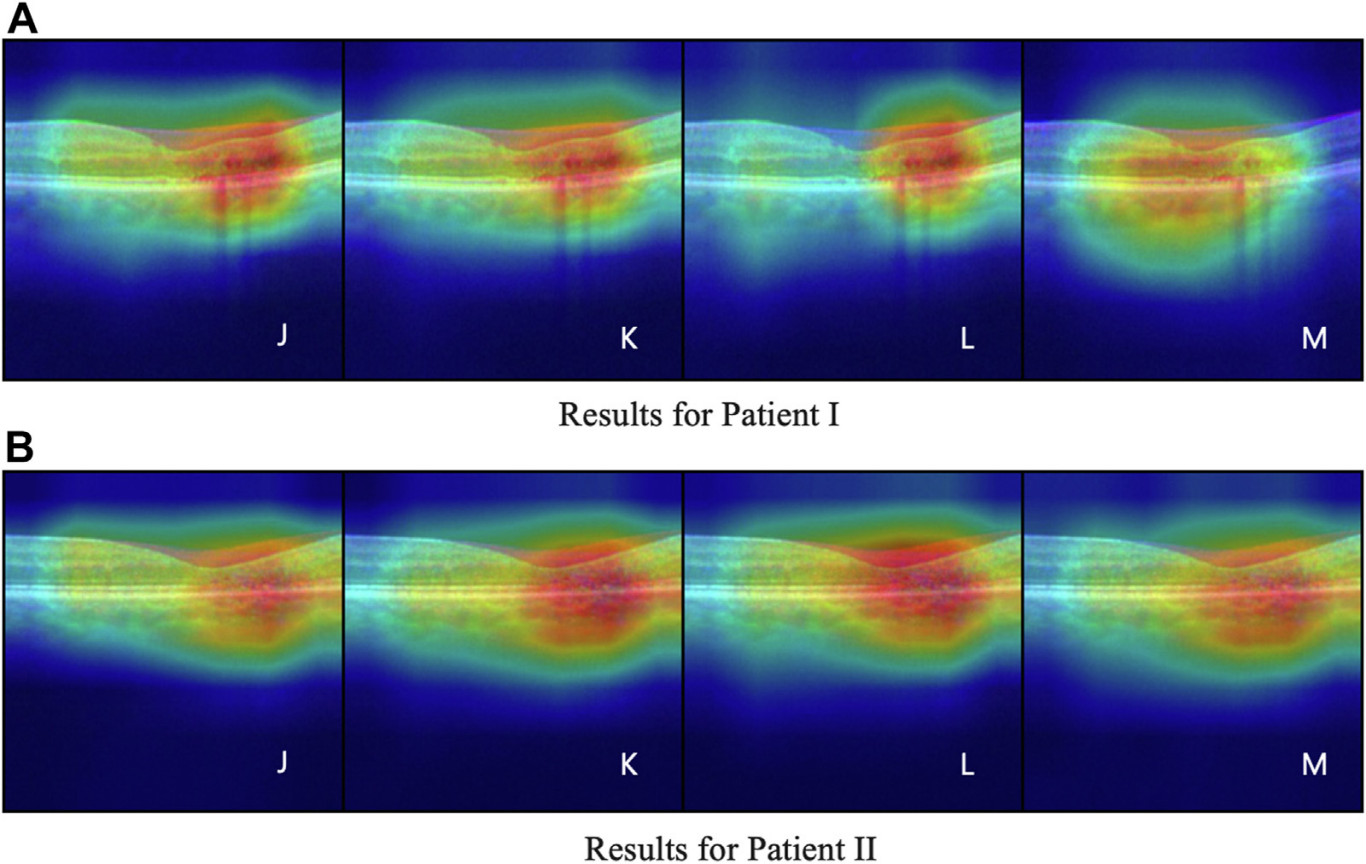
Grad-CAM results for all ensemble models for 2 patients with MacTel are shown in panels **A** (Patient 1) and **B** (Patient 2), when different combinations of individual models are used in ensemble based on uniform averaging. Image tags are aligned with the model tags in Table 3 where J–M show the results when we change the amount of labeled data used in training from 100% to 10%. Grad-CAM = gradient-weighted class activation maps; MacTel = macular telangiectasia.

**Figure 4:**
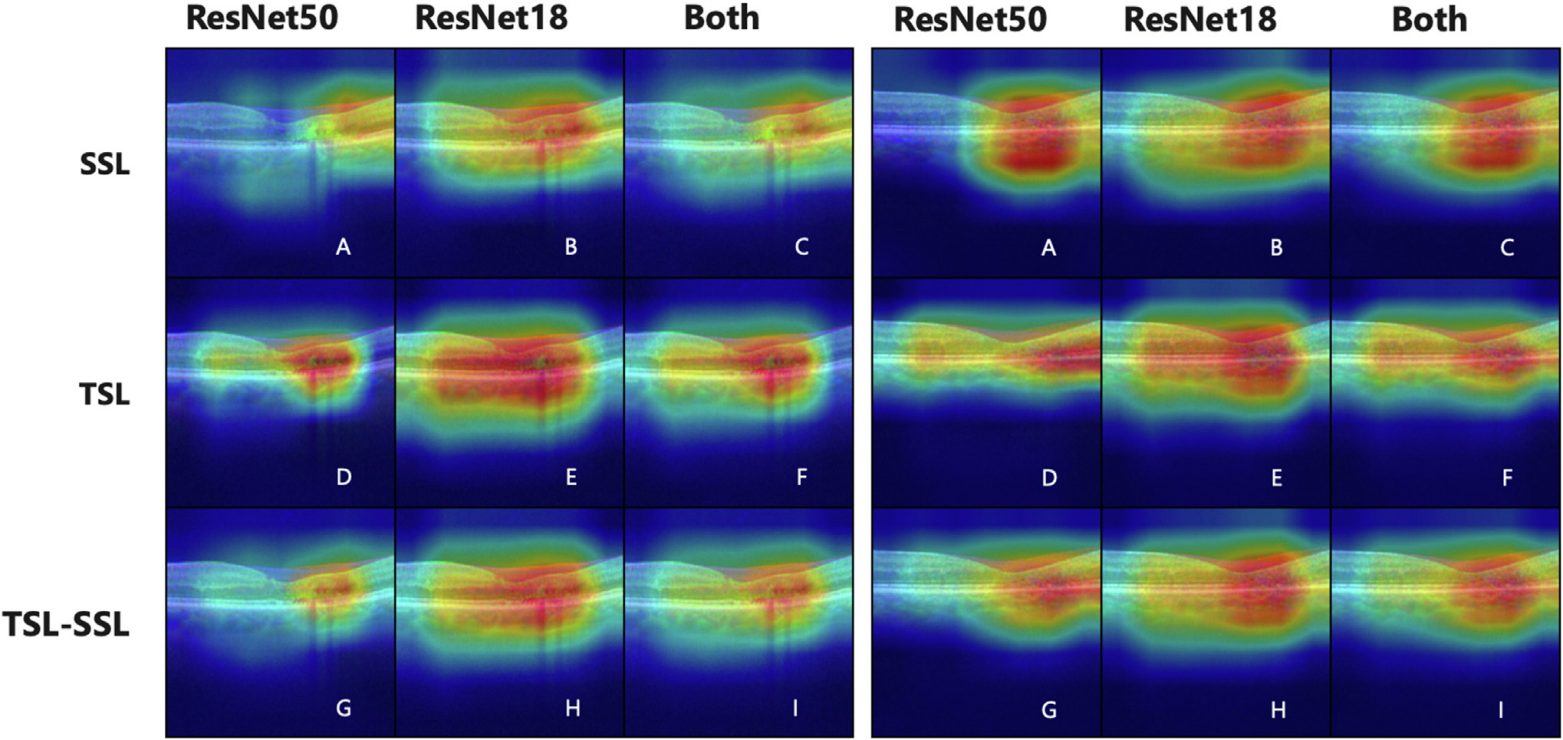
Grad-CAM results for all ensemble models for 2 patients with MacTel when different combinations of individual models are used in the ensemble based on uniform averaging. Image tags are aligned with the model tags in Table 3 where A–C show the results when individual models are trained based on the self-supervised learning (SSL) approach, D–F show similar results for training based on the traditional supervised learning (TSL) approach, and G–I show the cases where individual models trained based on TSL and SSL approaches are used in the ensembles. The column headers show the architecture used in the ensemble. Grad-CAM = gradient-weighted class activation maps; MacTel = macular telangiectasia.

We conduct a qualitative analysis of the Grad-CAM results, as shown in Figure 1, Figure 3, Figure 4. Compared with the individual models, the ensemble approach yields more focused results on the pathology of interest.

## Discussion

We present an ensemble-based approach based on deep learning models for accurate and interpretable detection of MacTel type 2 from OCT scans. Our experimental results clearly demonstrate improvements in both MacTel classification accuracy and interpretability when compared to the use of individual models. We found that ensemble models exhibited superior agreement with the assessments of the most experienced human experts, as well as the ensemble of human experts.

One of the key advantages of our approach lies in its ability to harness the power of SSL and ensemble techniques in the context of medical image analysis, particularly for diagnosing rare diseases like MacTel. The use of an ensemble method effectively combines multiple deep learning models, each trained with varying architectures and learning paradigms, resulting in a unified and highly performant model. This ensemble approach notably enhances the accuracy and interpretability of MacTel classification, even with limited labeled data for model training.

Our study demonstrates that ensemble models can achieve comparable performance to human experts, showcasing the potential of machine learning in augmenting medical expertise. When comparing the performance of model M, trained only on 10% of the data, as presented in Table 4, with that of its individual models A, E, I, and M from Table 3, a noteworthy observation emerges. Despite having access to only 419 OCT volumes, which include 185 MacTel patients within the 10% labeled training dataset, the ensemble model demonstrates a level of performance that is comparable to that achieved by the ensemble of human expert grader assessments. This achievement is particularly noteworthy given the inherent difficulty of assembling a large number of expert graders in clinical scenarios. Additionally, model M surpasses the performance of less experienced graders G3 and G4 in both accuracy and specificity. The ensemble model performance stands in contrast to the individual models, which do not exhibit the same level of performance when used separately.

Model J trained on the entire training dataset in Table 4 emerged as the top performer in our study. This expansive ensemble leverages the collective strengths of all the individual models, resulting in remarkable performance gains compared to the best-performing individual model G. While proper MacTel grading in practice requires a multimodal image set, model J surpasses all individual human graders, i.e., G1, G2, G3, and G4, by a notable 4% in terms of sensitivity, indicating its heightened ability to correctly identify MacTel cases by OCT alone, and manages to outperform the ensemble of human experts by a 1% margin in terms of sensitivity. Moreover, it achieves nearly identical performance as the ensemble of human experts in terms of AUROC, confirming its effectiveness in aligning with human expertise. However, it is important to note that we observed variations in the performance of the individual human graders. While the ensemble of human experts serves as a valuable reference, it exhibits some variability due to differing levels of expertise among graders. Based on the Cohen kappa matrix shown in Figure 2, interrater agreement varies between 0.74 and 0.95 among individual graders and the ensemble of human experts. Notably, 2 of our human graders possess extensive experience in evaluating this rare disease, with 14 to 17 years of MacTel grading expertise, a depth of knowledge that may be challenging to replicate in many clinical settings. This underscores the potential of our model to augment and complement the diagnostic capabilities of even the most seasoned ophthalmologists. Nevertheless, our ensemble model, particularly model J, demonstrates its potential as a powerful diagnostic tool that can compete effectively against a range of human experts, offering consistent and reliable performance in the diagnosis of rare diseases like MacTel.

Our approach contributes to the field of medical image analysis by addressing the critical issue of interpretability. Utilizing Grad-CAM to visualize the regions of interest within OCT images provides insights into the decision-making process of the models, which may help researchers to refine algorithms, improve diagnoses, and serve as a means to validate the models' predictions while gaining a deeper understanding of the underlying biological mechanisms.^20^, ^21^, ^22^, ^23^

Our visualizations reveal that specific regions of the retina affected by MacTel consistently capture the model's focus, indicating their significance in the diagnostic process. These results demonstrate the effectiveness of the ensemble approach in enhancing the localization of the target pathology. This transparency is vital in health care applications, enabling clinicians to understand and trust the model's predictions. However, it is important to recognize that these techniques establish correlation rather than causation. Furthermore, the interpretability provided by Grad-CAM, while valuable, serves as a visual aid, and future work may explore more advanced explainability techniques to address this limitation.

Our approach is not without limitations. One primary limitation is the requirement for labeled data, which is often scarce for rare diseases. Moreover, the quality and consistency of labels can vary between human graders, potentially introducing biases into the training process. Another limitation is the computational cost associated with training and utilizing multiple deep learning models in an ensemble. Training numerous models with different architectures and learning paradigms demands significant computational resources and time. This can be a barrier for institutions or researchers with limited access to high-performance computing resources. Additionally, while our ensemble approach improves performance, it is not a replacement for expert medical judgment. Clinicians should always exercise their expertise in conjunction with machine learning tools to make informed decisions in patient care.

The combination of SSL and model ensemble approaches improves accuracy and interpretability of automated OCT-based diagnosis of MacTel, a rare disease with limited labeled training data. The combination of quantitative metrics, qualitative visualizations, and comparison to human expert grading enhances the overall reliability of our approach. This approach has the potential to be a valuable tool for assisting health care professionals in the diagnosis of MacTel, as well as for similar image-based automated diagnostic models for other rare diseases with similar training data constraints.

Exploring different weighting methods and ensemble techniques offers a promising avenue for future research in medical image analysis. Variations in ensemble composition and weighting strategies, such as adaptive boosting,^24^ stacked generalization,^25^, ^26^, ^27^ or soft-voting methods,^28^ could provide insights into maximizing diagnostic performance, particularly in challenging or imbalanced datasets. By refining how models are combined or emphasizing certain predictions, future studies could further optimize ensemble models to improve classification accuracy, reliability, and generalizability across various clinical applications. This approach could ultimately enhance the robustness of automated diagnostic tools and support more nuanced decision-making in clinical practice.
