# Supplementary material for: Enhanced Macular Telangiectasia Type 2 Detection: Leveraging Self-Supervised Learning and Ensemble Models
Source: Ophthalmol Sci. 2025 Jan 13;5(4):100710. doi: 10.1016/j.xops.2025.100710 (PMC11987621; doi:10.1016/j.xops.2025.100710)
Supplement: Supplemental Appendix [file mmc1.pdf]

Supplementary Material

Supplemental Methods

AdaBoost (Adaptive Boosting) is an ensemble learning method that combines multiple individual classifiers to build a strong classifier. DL-AdaBoost extends this idea to incorporate deep learning models, which aims to improve the generalization performance by emphasizing difficult examples.[7] For a more detailed understanding, the algorithmic flow of DL-AdaBoost is depicted in Figure S5 and explained below, illustrating the sequential nature of classifier training and the adaptation of weights. At each iteration, the sample distribution is updated to emphasize challenging samples. The algorithm terminates when the weighted error becomes convergent and reaches a stable state. The outputs of DL-AdaBoost are the learned weights along with the final integrated model, which is a weighted combination of the individual models. The weights assigned to each model, denoted as α, reflect their contribution to the ensemble. To aggregate the predictions, we utilized α as weights to combine the predicted probabilities generated by the deep learning models. The algorithm includes the following steps:

1. **Input:**
   This step specifies the input to the algorithm, which includes labeled data (x_i, y_i), where x_i represents the feature vector and y_i represents the corresponding label for each data point. It also includes the hypothesis models h_t for $t = 1, \ldots, T$, which are the individual classifiers built based on a portion of labeled data and a chosen architecture.

2. **Initialize:**

   Initialize the distribution of samples $D_1(i) = 1/m$, where m is the total number of samples. This distribution represents the weights assigned to each sample, initially set uniformly.

3. **Repeat-Until Loop:**

   The algorithm iterates until a stopping criterion is met, which typically involves the weighted error reaching convergence.

4. **For Loop:**

   Within each iteration, iterate over each individual classifier h_t.

5. **Select hypothesis model h_t:**

   Choose an individual classifier h_t from the set of available hypothesis models.

6. **Compute weighted error ϵ:**

   Calculate the weighted error of the chosen individual classifier, which is the probability of misclassification weighted by the distribution of samples D_t.

7. **Compute weight α:**

   Calculate the weight assigned to the individual classifier h_t based on its weighted error. The weight α is computed using the formula $\alpha = \frac{1}{2}\ln\left(\frac{1-\epsilon_t}{\epsilon_t}\right)$.

8. **Update distribution D_{t+1}:**

   Update the distribution of samples $D_{t+1}(i)$ for the next iteration using the formula provided. This formula adjusts the weights of samples based on their misclassification by the individual classifier h_t.

9. **Output:**

   Once the loop converges (weighted error reaches convergence), output the weights α assigned to each individual classifier and the final model $H(x)$, which is the weighted sum of all individual classifiers.

DL-AdaBoost Results:

DL-AdaBoost iteratively finds weights to assign to individual classifiers based on their performance, and combines them to build a strong classifier. The differential learning aspect emphasizes difficult examples by adjusting the sample distribution during each iteration. Similar to the results for uniform averaging of weights presented in this study, we constructed 17 different ensemble models using various combinations of the individual models to assess the impact of each model training configuration based on DL-AdaBoost technique. The first set of ensembles was formed based on individual models trained via the TSL approach. They are named RESNET50-ADB(TSL), RESNET18-ADB(TSL), and RESNET(50&18)-ADB(TSL) based on the architecture(s) and methods used for model training. For example, in RESNET50-ADB(TSL), only individual models, which are trained based on the ResNet50 architecture, utilize the DL-AdaBoost algorithm for ensembling. RESNET(50&18)-ADB(TSL) incorporates a combination of ResNet18 and ResNet50 architecture-based models. Note that in naming the models, we used RESNET(50&18) to indicate the case where individual models based on both architectures are used in the ensemble. The second set of ensembles is similar to the previous set except that we used individual models based on the SSL approach. The third set of ensembles is formed using models trained based on SSL and TSL approaches. In the fourth set of ensembles, we fix the percentage of labeled training data used to train individual models based on different architectures

and learning paradigms. This case is specifically important in the context of rare diseases when limited data is available for model training. For the final set of models, we split the training data into four non-overlapping subsets and independently trained models using either SST or TSL for a single architecture choice. We then created ensembles from these models. "4X" indicates that each ensemble consists of four independently trained models, each trained on 25% of the training data.

Table S6 presents the weights learned for each ensemble. These weights signify the importance assigned to each individual model within the ensemble. The accuracy results are shown in Tables S7 and S8. Cohen’s Kappa Matrix reflects on the inter-rater agreements based on the DL-AdaBoost algorithm as demonstrated in Figure S6. We also combined Grad-CAMs of the individual models based on the learned weights to capture the collective contributions of the individual models in the ensemble as shown in Figures S7 and S8. We observed no considerable difference in accuracy metrics between DL-Adaboost and uniform averaging and the results are aligned with the uniform averaging method presented in this study.

Table S6. Weights learned by DL-AdaBoost for each individual model used in each ensemble: The first part of the models' names indicate the architectures used for models included in each ensemble and training approach is shown in parentheses.

TAG	Architecture		RESNET50				RESNET18			
	% of Labels		10%	25%	50%	100%	10%	25%	50%	100%
A	RESNET50-ADB(TSL)	TSL:	0.231	0.274	0.210	0.285	-	-	-	-
B	RESNET18-ADB(TSL)	TSL:	-	-	-	-	0.170	0.224	0.275	0.332
C	RESNET(50&18)-ADB(TSL)	TSL:	0.130	0.158	0.110	0.149	0.072	0.096	0.130	0.155
D	RESNET50-ADB(SSL)	SSL:	0.144	0.135	0.614	0.144	-	-	-	-
E	RESNET18-ADB(SSL)	SSL:	-	-	-	-	0.380	0.095	0.474	0.380
F	RESNET(50&18)-ADB(SSL)	SSL:	0.025	0.170	0.493	0.203	0.356	0.188	0.434	0.356
G	RESNET50-ADB(SSL-TSL)	SSL:	0.125	0.163	**0.503**	0.197	-	-	-	-
		TSL:	0.278	0.279	0.219	0.278	-	-	-	-
H	RESNET18-ADB(SSL-TSL)	SSL:	-	-	-	-	0.349	0.123	**0.426**	0.401
		TSL:	-	-	-	-	0.331	0.219	0.282	0.331
I	RESNET(50&18)-ADB(SSL-TSL)	SSL:	0.025	0.031	**0.070**	0.041	0.037	0.158	0.258	0.236
		TSL:	0.019	0.022	0.016	0.021	0.010	0.013	0.018	0.024
J	RESNET(50&18)-ADB(SSL-TSL)-100%	SSL:	-	-	-	0.237	-	-	-	**0.321**
		TSL:	-	-	-	0.287	-	-	-	0.154
K	RESNET(50&18)-ADB(SSL-TSL)-50%	SSL:	-	-	-	0.233	-	-	-	**0.345**
		TSL:	-	-	-	0.260	-	-	-	0.162
L	RESNET(50&18)-ADB(SSL-TSL)-25%	SSL:	-	-	-	0.237	-	-	-	**0.362**
		TSL:	-	-	-	0.215	-	-	-	0.186
M	RESNET(50&18)-ADB(SSL-TSL)-10%	SSL:	-	-	-	0.238	-	-	-	**0.293**
		TSL:	-	-	-	0.239	-	-	-	0.230

Table S7. Test set performance of ensembles based on DL-AdaBoost algorithm; the first part of the models' names indicates the architectures used in each ensemble. (50&18) shows that both architectures are used in the ensemble. Training approaches used for individual models are shown in parentheses. The % number in the models' names shows the particular percentage of labeled data used in the training of individual models.

TAG	ENSEMBLE MODEL	AUROC (CI 95%)	AUPRC (CI 95%)	ACCURACY	SENSITIVITY	SPECIFICITY
A	RESNET50-ADB(TSL)	0.964 (0.963, 0.966)	0.963 (0.961, 0.965)	0.915	0.900	0.927
B	RESNET18-ADB(TSL)	0.968 (0.966, 0.969)	0.960 (0.958, 0.962)	0.919	0.910	0.926
C	RESNET(50&18)-ADB(TSL)	0.976 (0.974, 0.977)	0.973 (0.972, 0.975)	0.923	0.903	0.939
D	RESNET50-ADB(SSL)	0.970 (0.969, 0.972)	0.968 (0.966, 0.970)	0.904	0.950	0.867
E	RESNET18-ADB(SSL)	0.976 (0.975, 0.977)	0.971 (0.969, 0.972)	0.912	0.961	0.872
F	RESNET(50&18)-ADB(SSL)	0.977 (0.976, 0.978)	0.973 (0.971, 0.974)	0.911	0.959	0.872
G	RESNET50-ADB(SSL-TSL)	0.978 (0.977, 0.979)	0.977 (0.976, 0.978)	0.935	0.947	0.925
H	RESNET18-ADB(SSL-TSL)	0.977 (0.976, 0.978)	0.975 (0.973, 0.976)	0.909	0.950	0.877
I	RESNET(50&18)-ADB(SSL-TSL)	0.979 (0.978, 0.980)	0.978 (0.976, 0.979)	0.924	0.960	0.894
J	RESNET(50&18)-ADB(SSL-TSL)-100%	**0.980 (0.979, 0.981)**	**0.980 (0.979, 0.981)**	0.933	0.942	0.926
K	RESNET(50&18)-ADB(SSL-TSL)-50%	0.979 (0.978, 0.981)	0.978 (0.976, 0.979)	0.928	0.966	0.896
L	RESNET(50&18)-ADB(SSL-TSL)-25%	0.979 (0.978, 0.980)	0.977 (0.975, 0.978)	0.929	0.939	0.921
M	RESNET(50&18)-ADB(SSL-TSL)-10%	0.974 (0.973, 0.975)	0.969 (0.967, 0.971)	0.925	0.916	0.933
N	HUMAN EXPERT ENSEMBLE	0.977 (0.975, 0.978)	0.987 (0.986, 0.987)	0.968	0.929	1

Table S8. Test set performance of ensembles based on DL-AdaBoost; the first part of the models' names indicates the architectures used in each ensemble. Training approaches used for individual models are shown in parentheses. The % number in the models' names shows the particular percentage of labeled data used in the training of individual models. 4X means 4 independently trained models based on 25% of training data are used in ensembles.

**Tag**	**Ensemble Model**	**AUROC (CI 95%)**	**AUPRC (CI 95%)**	**Accuracy**	**Sensitivity**	**Specificity**
O	RESNET50-4X(SSL)-25%	0.967 (0.966, 0.969)	0.964 (0.963, 0.966)	0.911	0.935	0.893
P	RESNET50-4X(TSL)-25%	0.973 (0.971, 0.974)	0.971 (0.970, 0.973)	0.932	0.96	0.91
Q	RESNET18-4X(SSL)-25%	0.978 (0.977, 0.979)	0.976 (0.975, 0.978)	0.921	0.965	0.885
R	RESNET18-4X(TSL)-25%	0.973 (0.972, 0.974)	0.969 (0.967, 0.970)	0.921	0.91	0.93
**L**	**RESNET(50&18)-ADB(SSL-TSL)-25%**	0.979 (0.978, 0.980)	0.977 (0.975, 0.978)	0.929	0.939	0.921

Algorithm 1 DL-AdaBoost

Input: (x_i, y_i) where $i = 1, ..., m$, x_i is the feature vector, and y_i is the label of x_i, hypothesis models h_t for $t = 1, ..., T$, built based on a portion of labeled data and choice of architecture

Initialize: distribution of samples $D_1(i) = \frac{1}{m}$

repeat

  for $t = 1$ **to** T **do**

    Select hypothesis model h_t

    Compute weighted error $\epsilon = \mathbb{P}[h(x) \neq y]$

    Compute weight $\alpha = \frac{1}{2} \ln \left(\frac{1-\epsilon_t}{\epsilon_t} \right)$

    Update $D_{t+1}(i) = \frac{D_t(i) \exp(-\alpha y_i h_t(x_i))}{Z_t}$;

    where Z_t is the normalization factor

  end for

until weighted error is convergent

Output: weights α, final model $H(x) = \sum_{t=1}^{T} \alpha_t h_t(x)$

Figure S5. DL-AdaBoost algorithm

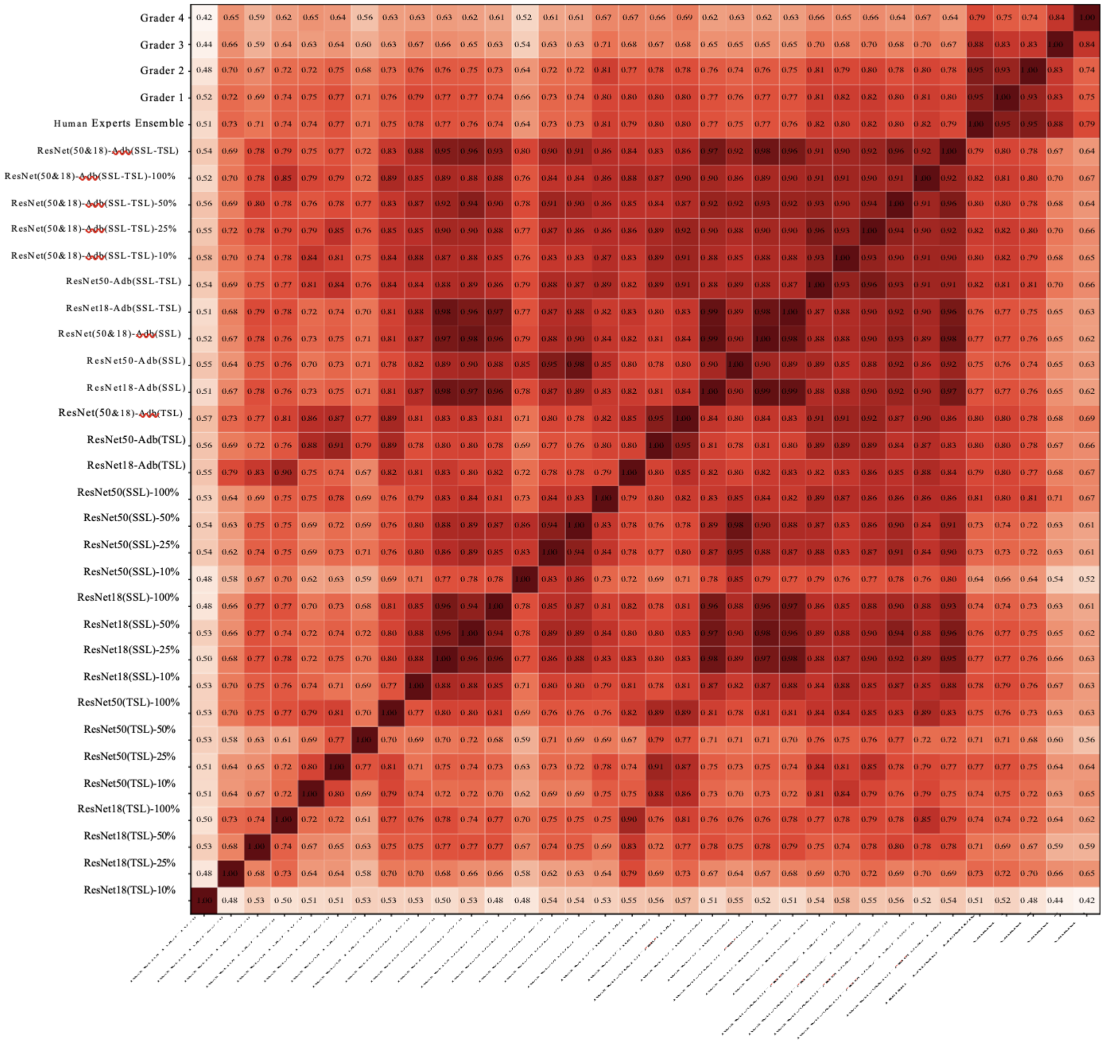

Figure S6. Cohen's Kappa Matrix reflects on the inter-rater agreements based on the DL-AdaBoost algorithm. We compare agreement between deep learning individual and ensemble models as well as graders. Grader 3 and 4 have less agreements with other graders and deep learning models due to less experience with grading OCT images for MacTel. Models trained based on self-supervised learning show better alignment with the most expert graders, i.e., graders 1 and 2. Ensemble models show better agreement to human expert graders than individual deep learning models.

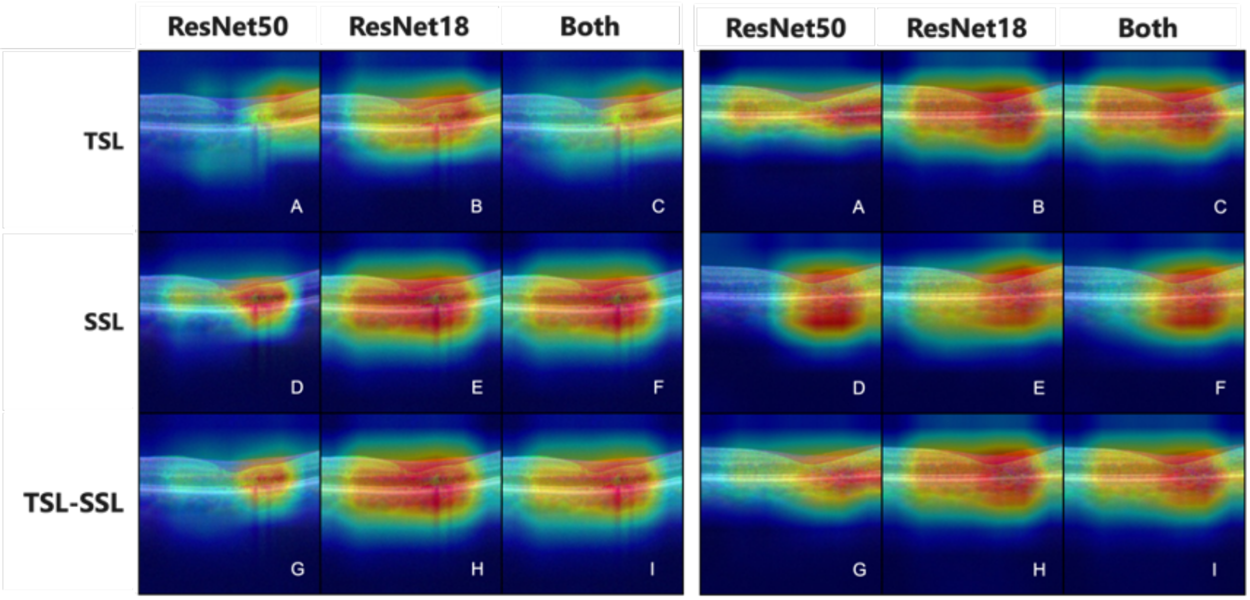

Figure S7. Grad-CAM results for all ensemble models based on the DL-AdaBoost algorithm for two patients with MacTel when different combinations of individual models are used in Adaboost. Image tags are aligned with the model tags in Table 3 where A-C show the results when individual models are trained based on the TSL approach, D-F show similar results for training based on the SSL approach, and G-I show tha cases where individual models trained based on TSL and SSL approaches are used in the ensembles. The column headers show the architecture used in the ensemble.

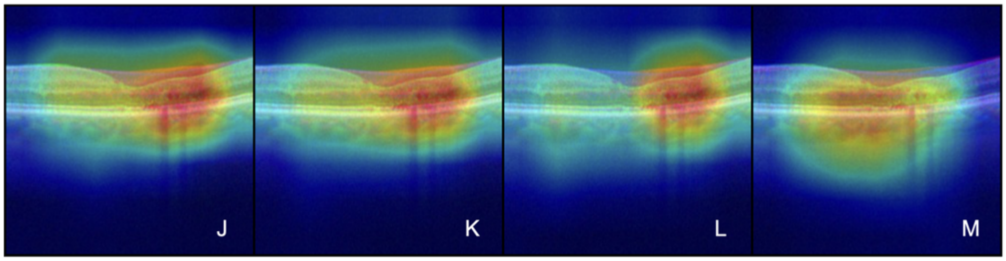

(a) Results for patient I

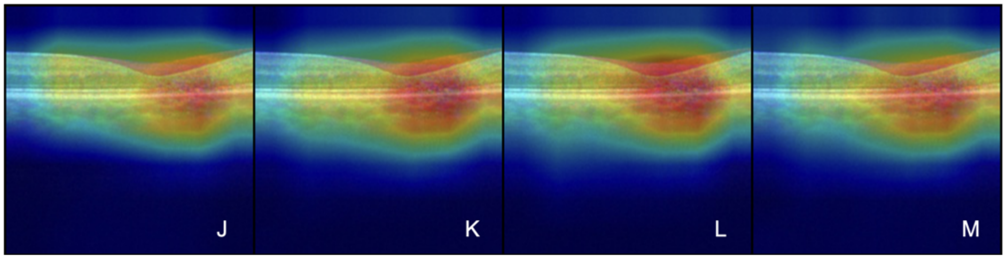

(b) Results for patient II

Figure S8. Grad-CAM results for ensemble models based on the DL-AdaBoost algorithm for two patients with MacTel when only a single percentage of the labeled data is used for training the individual models. Image tags are aligned with the model tags in Table 3 where J-M show the results when we change the amount of labeled data used in training from 100% to 10%.
